# Surgery guided by mixed reality: presentation of a proof of concept

**DOI:** 10.1080/17453674.2018.1506974

**Published:** 2018-10-23

**Authors:** Thomas M Gregory, Jules Gregory, John Sledge, Romain Allard, Olivier Mir

**Affiliations:** a Department of Orthopaedic Surgery, Avicenne Teaching Hospital, Assistance Publique—Hôpitaux de Paris, University Paris-Seine-Saint-Denis, Sorbonne Paris Cité, Bobigny, France;; b Moveo Institute, University Paris-Seine-Saint-Denis, Sorbonne Paris Cité, Bobigny, France;; c Department of Radiology, Beaujon Teaching Hospital, Assistance Publique—Hôpitaux de Paris, University Paris-Diderot, Clichy, France;; d Department of Orthopedic Surgery, Lafayette Hospital, Lafayette, LA, USA;; e Department of Ambulatory Care, Gustave Roussy Cancer Campus, Villejuif, France

Surgical innovation aims to improve both patient outcome and safety. Among many others, computer-based solutions such as 3-D anatomical reconstruction and computer-based procedures planning have shown clear benefits to patients (Beckmann et al. 2009, Hernandez et al. 2017, Saragaglia et al. 2018). The next step in the usage of 3D anatomical information is to go from planning to per-operative usage. This can be done using augmented reality (AR), a technique that allows the superimposing of a digital image on top of the visual field, i.e., augmenting reality. As virtual elements in the form of holograms align with the reality this is commonly referred to as mixed reality (MR) as it enhances reality.

Recently, Microsoft has developed a new concept of an MR headset (HoloLens), equipped with motors, that allows the hand user to interact with the headset by oral command or simple gesture.

The promise of such technology in the surgical field is huge, as it allows the surgeon (i) to gain access to computer-based solutions in real time during the procedure while remaining totally sterile, (ii) to gain access to 3-D holograms related to the patient imaging or the surgical technique, and (iii) to remotely interact with colleagues located outside the theatre.

Here, we present the first use of such a system in a surgical environment and focus on the latest trends of the rapidly developing connection between MR and surgery.

## Methods

### Patient

The patient was an 80-year-old woman (height 155 cm, weight 52 kg) with advanced arthritis of the shoulder combined with rupture of the tendons covering the joint, motivating the implantation of a shoulder prosthesis.

### Technical setup

The HoloLens headset is a self-contained, holographic computer, enabling its user to engage with her/his digital content and interact with holograms in the world around her/him. The technical characteristics of the device are depicted in the supplementary Appendix. No specific installation time in the operating room is required, the device being wireless.

### Data preparation

The raw DICOM files are too large to be downloaded into the headset. Therefore, the DICOM data stored in the hospital PACS were instantly loaded and preprocessed in a Cloud Unit (Azure Graphic Processing Unit—Microsoft Corp, Redmond, WA, USA). During the surgery, the DICOM data are made available for the headset through a dedicated radiological holographic application (TeraRecon Holoportal—https://www.terarecon.com/) connected via WiFi to the Cloud Unit.

### Surgical procedure

A standard reverse shoulder arthroplasty was performed. The challenge in this surgery was the limited bone stock of the vault (due to the patient’s anatomy with a Walch A2-type glenoid).

### Navigational technique

There was no calibration between HoloLens and the navigation system, as the MR headset has a capacity to drag the holograms manually. Subsequently, the holographic 3-D reconstruction of the scapula was manually positioned in such a way that the visible part of the bone matched with the corresponding part of the hologram. The surgeon was then able to see the hidden part of the scapula in a holographic mode.

### Funding and potential conflicts of interest

Funding source: MOVEO Foundation. Dr Mir has acted as a consultant for Amgen, BMS, Eli-Lilly, Lundbeck, MSD, Novartis, Pfizer, Roche, Servier and Vifor. Dr Gregory has acted as a consultant for Evolutis. The other authors have no conflict of interest to disclose.

## Results

**Figure 1.**
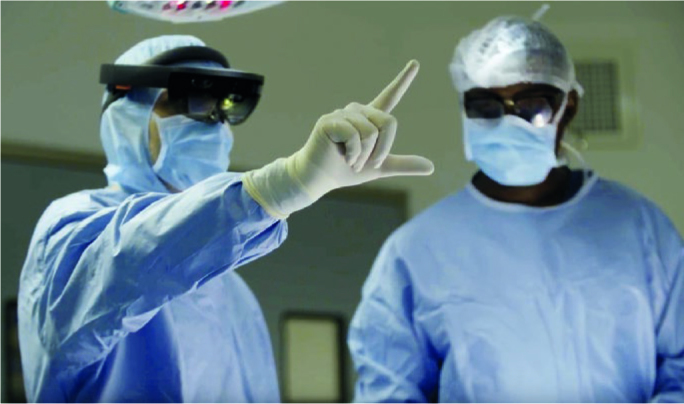
Outside view of the headset during the surgical procedure.

**Figure 2.**
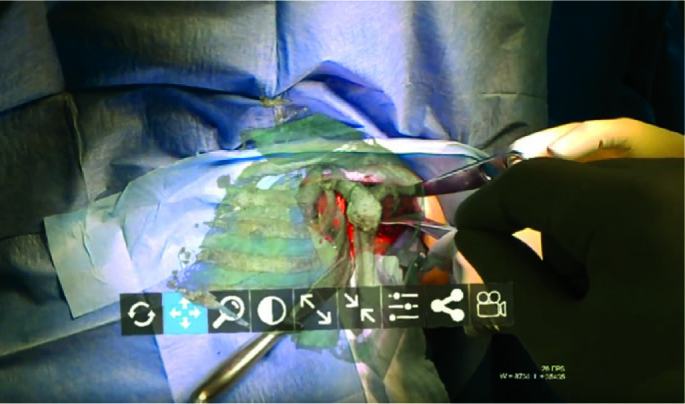
View from the headset during the surgical procedure.

The prosthesis was implanted with the aid of this MR headset at Avicenne Hospital in Bobigny, France, on December 5, 2017. As described above, the surgeon was able to access the patient’s medical data combined with the data of the operative technique, which were transmitted into his operating headset in real time during the intervention, e.g., patient scapula 3-D reconstruction extracted from her CT scan, the planning of the positioning of the glenoid and the whole operative technique developed in 3-D holograms according to the planned position. Hence, the surgeon was able to compare, stage by stage, what he was doing with what had to be done. The surgeon was also able to drag the 3-D reconstruction of the scapula, scaled 100%, right before his eyes, by simple gestures in front of the headset (Figure 1) in approximately 3 minutes. He was then able to superimpose the CT scan scapula reconstruction with manual adjustment onto the visible part of the bone (Figure 2 and see video at https://youtu.be/oQu1rGt6ym4).

The surgeon also shared what he saw in his headset with 4 other surgeons in the USA and in the UK, who were able to send information in the operator’s field of vision, via Skype, throughout the intervention (which was taking place in France). This is the first time that such complete usage of the immersive and collaborative aspects has been implemented in surgery of the shoulder.

The total duration of surgery was 90 minutes, similar to the mean duration of such a procedure in the operator’s experience (without the use of HoloLens). The duration of the procedure was prolonged due to the fact that it was broadcast on the Web and required dedicated time to comment on every step. On the other hand, a better understanding of the patient’s anatomy as allowed by the use of the headset probably contributed to time-saving.

**Figure 3.**
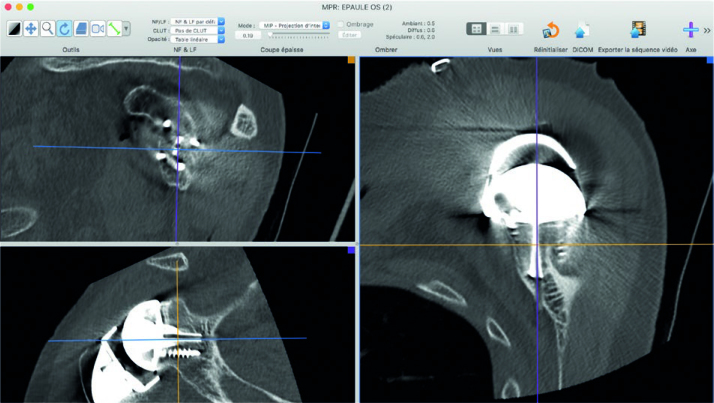
Postoperative CT scan of the glenoid (top left: sagittal view; bottom left: coronal view; right: axial view).

Postoperative CT scan showed adequate position of the prosthesis (Figure 3). The patient experienced no peri- or postoperative complication and was discharged home 3 days after surgery (similar to the routine practice in our institution).

Postoperative visit on day 45 confirmed the lack of postoperative complications.

## Discussion

Compared with a classical surgical procedure, the use of an MR headset might provide an improvement in outcomes for both surgeon and patient, without reducing the safety of the procedure. Indeed, thanks to 3-D holograms, the keyframes of the operation as well as critical organs (nerves, arteries…) are shown in real time by the surgeon. This provides a gain in accuracy and safety in the procedurem which finally might result in time-saving and correct positioning of the implant (Léger et al. 2017, Vávra et al. 2017). Notably, the technical setup of the device and data preparation before surgery had short duration (as explained above) but might be time-consuming for first-time users.

The improvement of the surgical procedure also lies in the opportunity for the surgeon to interact with colleagues and ask them for advice or warnings. The operation field is often small, and the surgeon’s gestures as well as an accurate view of the patient’s anatomy are restricted to only the surgeon and his assistant. Thanks to this new technology, the surgeon’s view is projected onto a screen, and accessible to many more people. This provides constant feedback on his/her decision, as well as being a potential educational tool.

The safety of the patient seems not be compromised by this new device. Indeed, the operating time was not lengthened, and no major adverse events occurred. On the contrary, the security of the surgery is probably improved, because the surgeon can see the patient’s anatomy directly, and other physicians can give helpful comments. As an illustration, Opolski et al. (2017) performed revascularization of a chronic coronary total occlusion assisted by AR glasses without adverse events.

This improvement does not incur a loss of comfort and focus. The weight of the device is low (579 grams, see supplementary Appendix), and the use of AR devices of comparable weight did not cause pain or tiredness (Sinkin et al. 2016, Léger et al. 2017). The amount of information is controlled in real time by the surgeon, so that the cognitive load is tolerable. The voice- and image-activated command is easy to use, without latency or excessive repetition (Léger et al. 2017). The image quality, contrast and stability are good enough not to cause tiredness or motion sickness. During the videoconferencing, no cut-offs occurred; thus, the constant of interaction and procedure safety was guaranteed. Finally, the battery has sufficient durability for most surgical procedures (up to 5.5 hours, see supplementary Appendix).

When a new technology appears, patient and staff approval is also a fundamental element. Muensterer et al. (2014) reported that their colleagues, staff, families and patients had a positive response to such technology.

Importantly, issues of data protection will have to be addressed for greater use of this technology (Muensterer et al. 2014, Vávra et al. 2017). AR interest in surgical procedures is strongly increasing, as shown by the large number of published studies. Reviews of the literature, randomized controlled trials, and single-center tests are multiplying (Muensterer et al. 2014, Sinkin et al. 2016, Léger et al. 2017, Opolski et al. 2017, Vávra et al. 2017, Pulijala et al. 2018). Various AR and MR devices are currently being tested, and data are convergeing toward the same results: procedure performance, and effectiveness as a learning tool. The divergence mainly involves surgeon comfort and satisfaction, and is related to the AR device itself (Table).

From an educational perspective, while the use of screens in the surgical theater has exploded over the last few years, the vision of high-quality AR for surgery (Ponce et al. 2014) or medical training has not yet been realized. To date, AR-based simulation is considered a promising approach in the surgical curriculum, allowing not only technical performance evaluation, but also telementoring.

Because the gap between research and wide application is mainly a question of cost, things could change rapidly in the next years thanks to cheaper AR devices. As mentioned in the ASiT position paper (Milburn et al. 2012), regional disparities in the availability of simulators and financial restraints are theoretical limitations to their wide use. We believe that recent, inexpensive devices will help lessen these expected difficulties. Importantly, dedicated teaching time should be made available within existing and future projects (Milburn et al. 2012).

In summary, we believe that our case report illustrates the fact that surgical practice and education can derive significant benefits from the implementation of AR and MR tools in daily practice. We believe that such an immersive and cost-effective approach would improve the training capacity of orthopedic surgery simulations.

**Table:** Current published data on AR in surgery

Study	Outcomes	Main findings
Vávra et al.^2017^	Review of the literature: augmented reality can presently improve the results of surgical procedures?	
		– AR is an effective tool for training and skill assessment of surgery residents, other medical staff, or students
		– The interest of surgeons is increasing
		– The performance is comparable to traditional techniques
		– The time required for completing a procedure has been reduced while using any form of AR
		– Inattentional blindness: the surgeon does not see an unexpected object which suddenly appears in his field of view
		– The amount of information is increasing and may be distracting
		– The latency of the whole system is also of concern
		– Long-term wear comfort is an issue
		– AR projections produce simulator sickness
Sinkin et al.^2016^	Ease of use	
		– Average ease of image capture =3.11/5
	Quality of images	
		– Average ease of video capture =3.22/5
	Gaze disruption	
		– Average ease of using the wink feature =1.89
	Distraction	
		– Quality of image and video =3.89 and 3.67/7
	Comfort and satisfaction to wear: Average for comfort =4.56/7, Average for satisfaction =3.78/7	
		– 33% of users felt the device to be a distraction from the case
Pulijala et al. 2017	Self-assessment scores of trainee confidence before and after the intervention. Novice surgical residents	
		– The study group participants showed greater perceived self-confidence levels
Opolski et al.^2017^	Operators’ satisfaction assessed by a 5-point Likert scale	
		– The voice-activated co-registration and review of images were feasible and highly rated by operators (4.7/5 points)
		– There were no major adverse events
		– More frequent selection of the first-choice stiff wire and lower contrast exposure
		– Success rates and safety outcomes remained similar between the two groups
Muensterer et al.^2014^	AR Glass was worn daily for 4 weeks to identify and evaluate daily activities with potential applicability	
		– Wearing Glass throughout the day was well tolerated
		– Colleagues, staff, families, and patients overwhelmingly had a positive response to Glass
		– Low battery endurance
		– Poor overall audio quality
		– Long transmission latency combined with interruptions and cut-offs during internet videoconferencing
Léger et al.^2017^	Time taken to perform the task. Attention shifts:	
		– There were significant reductions in terms of the time taken to perform the task, and attention shifts
	Feelings about accuracy, intuition, comfort, perceived cognitive load	
		– Users felt that the system was easier to use and that their performance was better
